# OTTM: an automated classification tool for translational drug discovery from omics data

**DOI:** 10.1093/bib/bbad301

**Published:** 2023-08-18

**Authors:** Xiaobo Yang, Bei Zhang, Siqi Wang, Ye Lu, Kaixian Chen, Cheng Luo, Aihua Sun, Hao Zhang

**Affiliations:** ShanghaiTech University; School of Life Science and Technology, ShanghaiTech University, 393 Huaxiazhong Road, Shanghai 200031, China; Shanghai Institute of Materia Medica; Drug Discovery and Design Center, Shanghai Institute of Materia Medica, Chinese Academy of Sciences, 555 Zuchongzhi Road, Shanghai 201203, China; University of Chinese Academy of Sciences, No.19A Yuquan Road, Beijing 100049, China; Beijing Proteome Research Center; State Key Laboratory of Proteomics, Beijing Proteome Research Center, and National Center for Protein Sciences (Beijing); Nanjing University of Chinese Medicine; School of Chinese Materia Medica, Nanjing University of Chinese Medicine, Nanjing 210023, China; Chemical Biology Research Center, Shanghai Institute of Materia Medica, Chinese Academy of Sciences, Shanghai 201203, China; academician medicinal scientist of the Chinese Academy of Sciences; Drug Discovery and Design Center, Shanghai Institute of Materia Medica, Chinese Academy of Sciences, 555 Zuchongzhi Road, Shanghai 201203, China; University of Chinese Academy of Sciences, No. 19A Yuquan Road, Beijing 100049, China; School of Life Science and Technology, ShanghaiTech University, 393 Huaxiazhong Road, Shanghai 200031, China; School of Chinese Materia Medica, Nanjing University of Chinese Medicine, Nanjing 210023, China; Shanghai Institute of Materia Medica; Chemical Biology Research Center, State Key Laboratory of Drug Research, Shanghai Institute of Materia Medica, Chinese Academy of Sciences, 555 Zuchongzhi Road, Shanghai 201203, China; University of Chinese Academy of Sciences, No. 19A Yuquan Road, Beijing 100049, China; School of Chinese Materia Medica, Nanjing University of Chinese Medicine, Nanjing 210023, China; Beijing Proteome Research Center; State Key Laboratory of Proteomics, Beijing Proteome Research Center, National Center for Protein Sciences (Beijing), Beijing Institute of Lifeomics; Research Unit of Proteomics-driven Cancer Precision Medicine, Chinese Academy of Medical Sciences; Shanghai Institute of Materia Medica; Chemical Biology Research Center, State Key Laboratory of Drug Research, Shanghai Institute of Materia Medica, Chinese Academy of Sciences, 555 Zuchongzhi Road, Shanghai 201203, China

**Keywords:** OTTM, omics data, translational drug discovery, literature mining, hepatocellular carcinoma

## Abstract

Omics data from clinical samples are the predominant source of target discovery and drug development. Typically, hundreds or thousands of differentially expressed genes or proteins can be identified from omics data. This scale of possibilities is overwhelming for target discovery and validation using biochemical or cellular experiments. Most of these proteins and genes have no corresponding drugs or even active compounds. Moreover, a proportion of them may have been previously reported as being relevant to the disease of interest. To facilitate translational drug discovery from omics data, we have developed a new classification tool named Omics and Text driven Translational Medicine (OTTM). This tool can markedly narrow the range of proteins or genes that merit further validation via drug availability assessment and literature mining. For the 4489 candidate proteins identified in our previous proteomics study, OTTM recommended 40 FDA-approved or clinical trial drugs. Of these, 15 are available commercially and were tested on hepatocellular carcinoma Hep-G2 cells. Two drugs—tafenoquine succinate (an FDA-approved antimalarial drug targeting CYC1) and branaplam (a Phase 3 clinical drug targeting SMN1 for the treatment of spinal muscular atrophy)—showed potent inhibitory activity against Hep-G2 cell viability, suggesting that CYC1 and SMN1 may be potential therapeutic target proteins for hepatocellular carcinoma. In summary, OTTM is an efficient classification tool that can accelerate the discovery of effective drugs and targets using thousands of candidate proteins identified from omics data. The online and local versions of OTTM are available at http://otter-simm.com/ottm.html.

## INTRODUCTION

Target-based drug discovery is the predominant strategy in pharmaceutical academia and industry [1]. Drug targets are typically identified through comprehensive omics data analysis and experimental validation [2]. The starting point for target discovery and validation largely depends on omics data in biomedical databases, which reduces the experimental workload by identifying correlations between a given disease and potential targets [3, 4]. On the one hand, the advancement of omics-driven biotechnology increases the reliability of the sources of evidence for disease-target relationship [5], accelerating the identification of new drug targets at the levels of genomics, transcriptomics, proteomics, and other omics technologies [6]. The analysis of differential protein or gene expression under physiological and pathological conditions also contributes to target discovery for specific diseases. On the other hand, an increasing number of FDA-approved and clinical trial drugs are available for efficacy assessment in translational medicine research. Exploring non-cancer drugs for anticancer activity has great potential for advancing novel therapeutics into clinical trials [7–9].

In our previous study, we collected and analyzed proteomics data from 110 paired tumor and non-tumor samples of early stage liver cancer related to hepatitis B virus infection. We identified sterol O-acyltransferase 1 (SOAT1) as a promising target for the discovery and development of drugs against hepatocellular carcinoma [10, 11]. However, the identification of SOAT1 from thousands of candidate proteins was accomplished via manual inquiry in drug–target databases and literature information, which limits the discovery of additional potential targets and drugs from the same batch of omics data. It is extremely difficult to explore the thousands of possibilities employing manual selection of candidate proteins as the starting point for subsequent validation. Thus, we sought to develop an automatic workflow and classification tool for identifying multiple targets and their potential drugs from one batch of omics data. Thousands of candidate proteins are overwhelming for subsequent experimental validation. Accordingly, the primary challenge lies in narrowing the range of candidate proteins from omics data sets.

We propose that this scale of possibilities can be substantially reduced via two steps, namely, drug availability assessment followed by literature mining [12, 13]. In step one, candidate proteins without corresponding drugs can be excluded from subsequent translational drug discovery [14]. Typically, hundreds or even thousands of differentially expressed proteins (DEPs) or genes (DEGs) are identified using omics approaches, some of which have corresponding FDA-approved or clinical trial drugs. Existing drug–target relational databases, such as DrugBank and the Therapeutic Target Database (TTD), provide comprehensive information on approved, clinical and investigational drugs or compounds associated with protein targets, which can be utilized to reduce the number of candidate proteins for further evaluation [15–17]. Step two involves literature mining for drugs or their corresponding target proteins that may have been previously reported as being relevant to the disease of interest. These target proteins and drugs can be excluded from further validation, thus further reducing the number of candidate proteins derived from omics data. These two steps substantially reduce the number of drugs and target proteins for subsequent experimental validation.

Here, we report the development of an automatic classification tool, which we named Omics and Text driven Translational Medicine (OTTM), that can efficiently accelerate translational drug discovery from omics data. OTTM was designed to facilitate the discovery of effective drugs as well as promising target proteins from omics data via drug availability assessment and literature mining. Our tool can greatly reduce the number of drugs and targets for subsequent experimental assessment. After drug availability assessment, candidate proteins with no corresponding drugs are excluded by OTTM, thus reducing the number of proteins that may merit further evaluation. Following the literature mining step, OTTM also excludes candidate proteins that have been previously reported as being associated with the disease of interest, which further reduces the number of proteins and drugs for experimental testing. Literature mining is undertaken by searching all available PubMed abstracts using designated keywords of disease type, such as hepatocellular carcinoma, lung carcinoma, atherosclerosis and diabetes. Finally, OTTM recommends multiple FDA-approved drugs or drugs undergoing clinical trials as well as their corresponding target proteins for subsequent translational drug discovery and further target validation.

## MATERIALS AND METHODS

### Data sources and processing

For drug availability assessment using OTTM, drug–target pair data (P1-01-TTD target table and P2-01-TTD drug table) were extracted from the TTD database [15, 16]. Human protein–protein interaction (PPI) data (9606.protein.physical.links.detailed.v11.5) were extracted from the STRING database [18] to construct the relevant candidate PPI protein list. Scores equal to or >700 were regarded as representing reliable PPIs. For literature mining, all PubMed abstract information was extracted from 1551 XML files (from pubmed22n0001 to pubmed22n1551). The table for mapping gene symbols to PubMed IDs was also extracted from the PubMed database. To improve the speed of text mining, all PubMed abstract information was saved to ElasticSearch using PubMed IDs as the index. The downloaded data and mapping tables used are detailed in Supplementary Table S1.

### Analysis of DEPs

The hepatocellular carcinoma proteome and transcriptome data sets were collected from the cohorts specified in Jiang *et al*. [10] and Gao *et al*. [19]. The Wilcoxon rank-sum test was used to identify DEPs and DEGs between tumor tissues and adjacent non-tumor tissues in each cohort. Jiang *et al* found that 3985 genes were upregulated (tumor: non-tumor ratio > 1, *P* < 0.05) in tumor tissues, as were their corresponding proteins, whereas 4327 genes were upregulated in tumor tissues in the cohort of Gao *et al*. A total of 4489 DEPs were obtained by merging the DEPs of the two cohorts and these were used as the candidate protein list for OTTM in this study. The *P*-values and ratios for these proteins are detailed in Supplementary Table S2.

### OTTM workflow

Based on the list of DEPs or DEGs provided by the user, OTTM classifies targets and recommends drugs in three steps. In the first step, proteins or genes without corresponding FDA-approved or clinical trial drugs are excluded. Based on the drug–target pair information in the TTD database, OTTM retains and categorizes only the target proteins that are associated with FDA-approved or clinical trial drugs. In the second step, OTTM excludes the proteins or genes that have been previously reported as being relevant to the disease of interest. If a target protein has not been reported as being relevant, its corresponding drug or drugs that were previously reported as being relevant are also excluded by OTTM. Accordingly, only the unreported drugs for unreported proteins are recommended for subsequent experimental testing. During literature mining, OTTM performs an exhaustive keyword search in all available PubMed abstracts using the keyword designated by the user. Typically, this keyword is the name of a disease, such as hepatocellular carcinoma, atherosclerosis, diabetes or, indeed, any other condition. If a target protein or drug and the designated keyword appear in the same PubMed abstract, the target protein or drug will be considered as previously reported and is excluded from further assessment. In the third step, after drug recommendation for every retained target protein, OTTM generates visualization charts for dozens of drugs with corresponding targets. These drugs are expected to be assessed in experimental testing.

### Exclusion of target proteins without available drugs

OTTM checks drug availability for every DEP or DEG provided by the user using internally stored drug–target pair information. Candidate proteins with available drugs are categorized into the ‘With Drug’ class by OTTM, whereas the other candidate proteins are categorized into the ‘No Drug’ class. OTTM then further classifies the targets with available drugs into three categories. Candidate target proteins with corresponding FDA-approved drugs are classified as ‘FDA Approved’; candidate protein targets with corresponding Phase1/2/3 clinical trial drugs are classified as ‘Clinical Trial’; and the candidate proteins that do not belong to either of these categories are classified as ‘Others’. In summary, the list of candidate proteins or genes provided by the user is categorized by OTTM into two classes according to drug availability and three subclasses according to drug status. These classifications are represented as pie charts by OTTM.

### Exclusion of previously reported targets and drugs

The relevance between a candidate protein and the disease of interest is assessed via literature mining using all available PubMed abstracts. OTTM searches every PubMed abstract for each candidate protein using a designated disease name as the keyword (e.g. hepatocellular carcinoma, atherosclerosis and diabetes). If the designated keyword is found in the same PubMed abstract as the candidate protein, then this protein is considered to be ‘previously reported as being relevant’ and is excluded from further assessment. In addition, for candidate proteins that have not been previously reported as being relevant, the relevance between their corresponding drugs and the disease of interest is also assessed by OTTM. Similarly, if the designated keyword and the drug name are found in the same PubMed abstract, then this drug is considered to be ‘previously reported as being relevant’ and is excluded from further assessment.

### Construction of the list of interacting proteins

Information for pairs of interacting proteins from the STRING database was used to construct a list of interacting proteins. Protein pairs with a combined score equal to or higher than 700 were regarded as physically interacting by OTTM. Among all the human proteins contained in the UniProt database, those that are not in the user-provided list are selected for further assessment. From these, all the proteins that physically interact with user-provided candidate proteins are retained for the construction of the interacting protein list. After assessment and construction, this list is evaluated by OTTM with its standard processes, including the two-phase target classification and drug recommendation. The user decides whether or not to test the recommended drugs relating to the list of interacting proteins depending on the number of recommended drugs associated with the DEPs. If enough drugs are recommended without considering the interacting proteins, users can choose to ignore them. However, the list of interacting proteins is constructed and evaluated by default.

### Drug recommendation for target proteins

For further assessment of experimental efficacy, OTTM automatically choose one drug as the recommended drug for each target protein. When several drugs correspond to the same target protein, OTTM recommends the drug with the highest frequency in the literature as it is the most likely to be available for purchase. In this scenario, the drug with the greatest number of relevant PubMed abstracts is given the highest ranking. However, the availability of recommended drugs is largely -->dependent on the region where the user is located and the suppliers of chemicals that a user has access to. Thus, users can choose alternative drugs belonging to the same target protein if the recommended drug is unavailable for purchase. When no drugs are available commercially for approximately half of the recommended target proteins, users should increase the number of recommended drugs in the configuration file and also consider including the recommended drugs associated with the interacting proteins.

### OTTM output types and visualization

OTTM outputs four types of results—a pie chart of target classification, a tree diagram of target classification, a sunburst chart of drug recommendations and tree diagrams of drugs for every target protein. These output HTML files are implemented with the ECharts scripts and can be easily visualized in typical internet browsers. For the target classification pie chart, the inner circle represents the statistics for proteins with or without drugs, whereas the outer circle shows the statistics for proteins with drugs of different statuses. Regarding the tree diagram of target classification, the first-level nodes are divided into FDA-approved targets and clinical trial targets, whereas the second-level nodes are divided into ‘Reported’ and ‘Not Reported’ targets. For the sunburst chart of drug recommendations, drugs and targets are shown in pairs, and the FDA-approved and clinical trial categories are shown in different colors. For each target protein, an ECharts HTML file showing all corresponding drugs is outputted. This HTML file includes a tree diagram showing the two-level drug classification and a bar plot showing the literature popularity of every corresponding drug.

### OTTM web server

OTTM is available both online and locally, starting from its web server (http://otter-simm.com/ottm.html). For online usage, users upload the list of candidate proteins or genes and the configuration file, which can determine the number of recommended drugs. Once finished, the results will be sent to users by e-mail, meaning that online users must provide an e-mail address in the configuration file. For local usage, users download the OTTM executable program package and necessary data files. Once properly configured, users can expect to get the results within several minutes of job submission on typical personal computers. The web server also provides a concise introduction of principles and a user manual containing detailed configuration and job submission instructions. As the input list of OTTM, one official symbol per line for each candidate protein or gene. Examples of a configuration file and a protein list are provided on the Search page.

### Cell viability assay

Analytically pure powders of the tested drugs were purchased from several companies. Detailed information is provided in Supplementary Table S7. The HepG2 and Huh7 cell lines were purchased from American Type Culture Collection (Manassas, VA, USA). The cells were cultured in 96-well plates at a density of 8000 cells per well in DMEM supplemented with 10% fetal bovine serum and 1% penicillin/streptomycin at 37°C with 5% CO_2_. After 24 h, the medium was replaced with fresh medium supplemented with various concentrations of compounds, and the cells were cultured for another 72 h. The CellTiter-Glo (CTG) Luminescent Cell Viability Assay was used to determine the viability of cells treated with drugs or control. Drugs were applied at the 50 and 100 μM concentrations with three replicates. After 72 h of incubation, CTG reagent was added to each well, and cell viability was determined using the ATP glow assay. Luminescence was measured using an EnVision (PerkinElmer) microplate reader. After screening, drugs with more than 50% inhibition at 100 μM were diluted to nine concentration gradients, and their half-maximal inhibitory concentration (IC_50_) values were measured using dose–response curve fitting. Cell viability data were analyzed using GraphPad Prism (version 8.0) software. Data are shown as means ± standard error of the mean.

**Figure 1:**
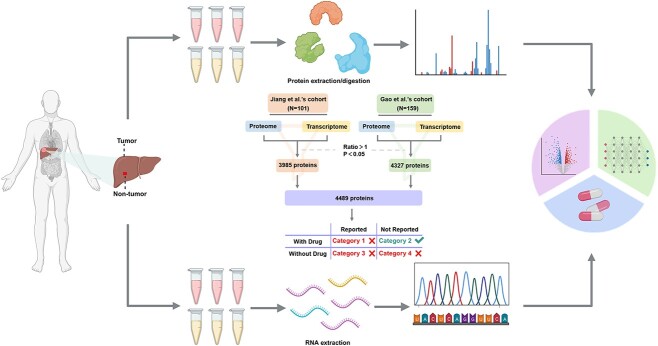
Proteins or genes from omics data are classified into four categories by OTTM. OTTM can classify thousands of DEPs or genes identified from omics data into four categories. Category 1: proteins have corresponding drugs but have been reported as being relevant to the disease of interest. Category 2: proteins have corresponding drugs and have not been reported as being relevant to the disease of interest. Category 3: proteins have no corresponding drugs and have been reported as being relevant to the disease of interest. Category 4: proteins have no corresponding drugs and have not been reported as being relevant to the disease of interest. OTTM aims to identify Category 2 proteins or genes from among all the proteins or genes identified from omics data.

## Results

### Purpose and principle of OTTM

In proteomic or transcriptomic studies using clinical samples, thousands of DEPs or DEGs are typically identified. Effective drugs and promising therapeutic targets are expected to be identified from these omics data. Ideally, these drugs should be FDA-approved or under clinical trial, and their corresponding target proteins should not have been previously reported as being relevant to the disease of interest. However, most candidate proteins or genes derived from omics data lack corresponding drugs or even compounds. Besides, some of these proteins or genes have already been reported in association with the disease of interest, and it is unnecessary to retest their corresponding drugs. Manual inquiry of drug availability and literature evidence for specific candidate proteins may lead to the discovery of one effective drug. However, it is not feasible to manually inspect every candidate protein identified from omics data. To efficiently and exhaustively address this question, we developed a new classification tool, named OTTM, which automatically recommends dozens of drugs from thousands of candidate proteins or genes, thus allowing for rapid experimental assessment and potential target discovery.

**Figure 2:**
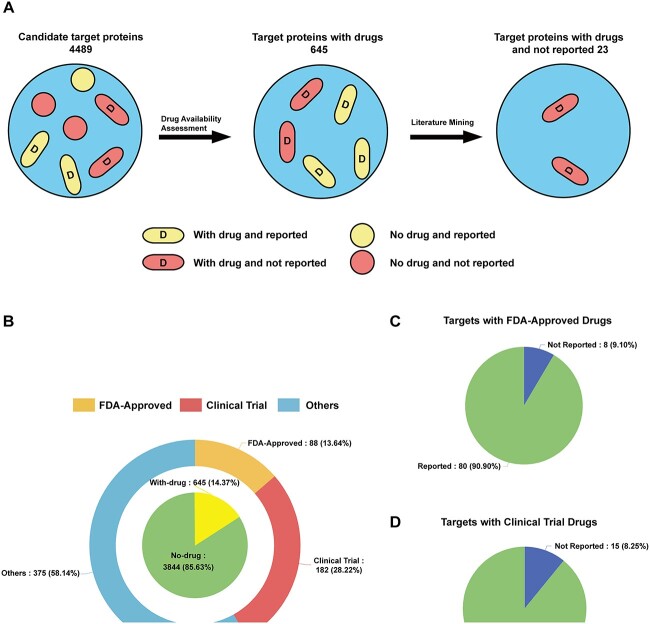
Schematic diagram of the principles involved in how OTTM classifies its target proteins via drug availability assessment. (**A**) Schematic illustration of how OTTM classifies target proteins from omics data. The yellow capsules with a capital ‘D’ represent target proteins that have corresponding drugs but have been previously reported as being relevant to the disease of interest. The red capsules with a capital ‘D’ represent target proteins that have corresponding drugs and have not been previously reported as being relevant to the disease of interest. The yellow and red circles represent target proteins without corresponding drugs. (**B**) From an initial 4489 DEPs, 645 target proteins with corresponding drugs were identified by OTTM. Of these, 88 (13.64%) are associated with FDA-approved drugs, 182 (28.22%) with drugs under clinical trial and 375 (58.14%) with drugs that have been discontinued. (**C**) Among the 88 target proteins associated with FDA-approved drugs, OTTM suggests that eight have not been reported as being relevant to hepatocellular carcinoma, whereas each of the other 80 is mentioned in at least one PubMed abstract that contains the designated keyword ‘hepatocellular carcinoma’. (**D**) Among the 182 target proteins associated with drugs under clinical trial, OTTM suggests that 15 have not been reported as being relevant to hepatocellular carcinoma, whereas each of the other 167 is mentioned in at least one PubMed abstract that contains the designated keyword ‘hepatocellular carcinoma’.

In OTTM, candidate proteins from omics data can be classified into four categories (Figure 1). Category 1 contains proteins that have corresponding drugs but have been previously reported in association with the disease of interest. This category can be classified as ‘With Drug and Reported’. Category 2 contains proteins that have corresponding drugs and have not been reported as being related to the disease of interest. This category can be classified as ‘With Drug and Not Reported’. Category 3 comprises proteins that have no corresponding drugs and have already been reported as being relevant to the disease of interest. This category can be classified as ‘Without Drug and Reported’. Category 4 encompasses proteins that have no corresponding drugs and have not been previously reported in association with the disease of interest. This category can be classified as ‘Without Drug and Not Reported’. Thus, OTTM is meant to identify all candidate proteins belonging to Category 2 among the proteins or genes from omics data. In general, OTTM excludes proteins from Categories 3 and 4 as candidates *via* drug availability assessment based on available drug–target pair information. Subsequently, OTTM excludes proteins in Category 1 from the remaining candidate proteins via literature mining using a designated disease name as the keyword. Finally, all proteins in Category 2 are retained for subsequent drug recommendation and experimental assessment (Figure 2A).

### Target classification *via* drug availability

For the 4489 DEPs identified from our previous omics study on hepatocellular carcinoma, OTTM indicated that 3844 (85.63%) have no corresponding drugs, whereas 645 (14.37%) do, as shown in the inner circle of the pie chart in Figure 2B. In the outer circle of the pie chart, the 645 target proteins with corresponding drugs are further classified according to the status of the corresponding drugs. Among the 645 candidate proteins with available drugs, 88 (13.64%) have corresponding FDA-approved drugs, 182 (28.22%) have corresponding drugs that are under clinical trial and 375 (58.14%) have corresponding drugs that have been discontinued owing to termination or failure of the respective clinical trials. After OTTM classification, it can be seen that more than 85% of candidate proteins have no corresponding drugs. Among the 645 target proteins with corresponding drugs, <42% candidate proteins have FDA-approved drugs or drugs undergoing clinical trials. In brief, only 270 target proteins (6.01% of the original 4489 candidate proteins) with FDA-approved or clinical trial drugs are retained by OTTM after drug availability assessment. Correspondingly, ~94% of candidate proteins are excluded from subsequent evaluation by OTTM owing to a lack of corresponding drugs.

### Target classification via literature mining

**Figure 3:**
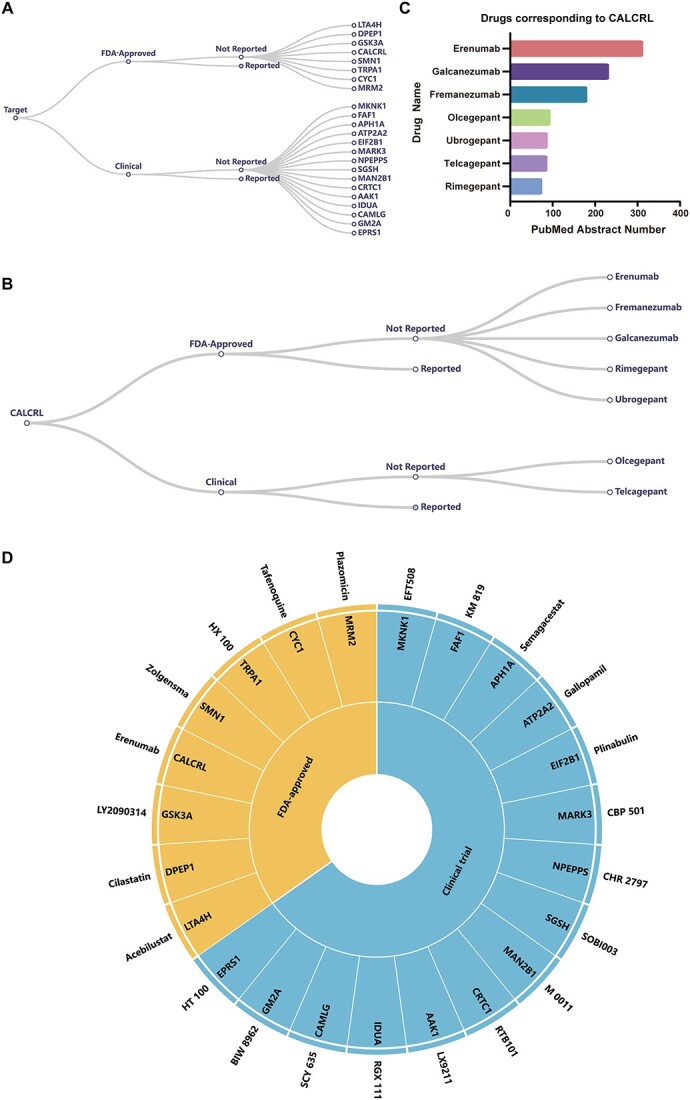
Drugs and target proteins recommended by OTTM for 4489 DEPs. (**A**) Target classification based on drug availability and literature mining in PubMed abstracts using ‘hepatocellular carcinoma’ as the designated keyword. OTTM suggested that 23 target proteins have not been reported as being relevant to hepatocellular carcinoma, including eight target proteins with FDA-approved drugs and 15 target proteins with drugs under clinical trial. Users can interactively click on each branch of this tree diagram to expand or collapse each category. (**B**) CALCRL is used as an example to illustrate the drug classification for a target protein. First, the drugs corresponding to a target protein are classified into FDA-approved and clinical trial categories. Then, these drugs are further classified into Reported and Not Reported categories based on the results of the literature mining using the designated keyword ‘hepatocellular carcinoma’. (**C**) Seven drugs corresponding to the target protein CALCRL were ranked by OTTM according to the number of PubMed abstracts containing their names. (**D**) From an initial 4489 candidate proteins, 23 drugs and 23 target proteins were recommended by OTTM for further experimental validation, including eight FDA-approved and 15 clinical trial drugs. One drug is recommended for each target protein.

Among the 270 target proteins with corresponding FDA-approved or clinical trial drugs, some have been previously reported in relation to hepatocellular carcinoma. To exclude these ‘Reported’ proteins, OTTM performs target classification via literature mining in all available PubMed abstracts using ‘hepatocellular carcinoma’ as the keyword. A target protein will be categorized by OTTM as ‘Reported’ if the keyword ‘hepatocellular carcinoma’ is found in any PubMed abstract mentioning this protein, and as ‘Not Reported’ if the keyword is not found in any PubMed abstract pertaining to this target protein. Among the 88 target proteins with corresponding FDA-approved drugs, eight have not been previously reported as being relevant to hepatocellular carcinoma; regarding the other 80 target proteins, each is mentioned in at least one PubMed abstract in conjunction with the designated keyword ‘hepatocellular carcinoma’ (Figure 2C). Among the 182 target proteins with corresponding drugs that are undergoing clinical trials, 15 have not been reported as being relevant to hepatocellular carcinoma, whereas 167 are mentioned in at least one PubMed abstract that also contains the designated keyword ‘hepatocellular carcinoma’ (Figure 2D). Briefly, after the above two phases of target classification, OTTM generates an ECharts tree diagram for all the 270 target proteins with corresponding drugs (Figure 3A), and these are retained by OTTM for subsequent drug recommendation and experimental testing. Users can interactively click on each branch of the tree diagram to expand or collapse each category.

Finally, OTTM performed drug availability assessment and literature mining and classified the initial 4489 candidate proteins into four categories. The names of the proteins in the four categories and their reported PubMed IDs are provided in Supplementary Tables S3–S6. Category 1 contains 542 proteins that have corresponding drugs but have been reported as being relevant to the disease of interest; Category 2 contains 103 proteins that have corresponding drugs and have not been reported as being associated with the disease of interest; Category 3 comprises 1763 proteins that have no corresponding drugs and have been reported as being relevant to the disease of interest; and Category 4 encompasses 2081 proteins that have no corresponding drugs and have not been reported in association with the disease of interest. Among the 103 proteins in Category 2, only 23 have corresponding FDA-approved drugs (8) or drugs under clinical trial (15). These 23 target proteins not only have corresponding FDA-approved or clinical trial drugs for subsequent efficacy evaluation but also have not been reported as being associated with hepatocellular carcinoma.

### Drug recommendations for each target protein

Each target protein may have more than one corresponding drug. However, to explore the potential correlation between a target protein and the disease of interest, it is not necessary to test all available drugs associated with one target protein. Thus, OTTM recommends one drug for each remaining target protein for subsequent experimental validation. OTTM generates an ECharts tree diagram for each remaining target protein, as shown in Figure 3B. The root node of the tree diagram is the name of the target protein and the second-level nodes are divided into FDA-approved drugs and clinical trial drugs. The third-level nodes are divided into ‘Reported’ and ‘Not Reported’ based on whether the drug name and the designated keyword appear in the same abstract. Users can interactively click on each branch of this tree diagram to expand or collapse each category.

OTTM recommends drugs based on their popularity in the literature, which is based on the frequencies of their appearance in all available PubMed abstracts. From the perspective of OTTM, drugs with greater literature popularity might have more detailed references, especially for subsequent efficacy assessment. For example, the target protein CALCRL has seven corresponding drugs, including five that are FDA-approved and two that are under clinical trial. Among these drugs, erenumab is top-ranking, with more than 300 mentions in relevant PubMed abstracts (Figure 3C). Accordingly, erenumab was the drug recommended by OTTM for the target protein CALCRL for subsequent evaluation. After drug recommendation for each target protein, OTTM generates an ECharts sunburst plot for all the remaining target proteins. The 23 target proteins remaining from the initial 4489 candidate proteins identified from omics data and their 23 corresponding drugs are shown in pairs (Figure 3D).

Notably, the drug recommended for a specific target protein might be unavailable for purchase, depending on the region where users are located. Users can replace this drug with an alternative one corresponding to the same target protein. Similarly, the drug recommended for a given target protein might be an antibody, and a small-molecule drug for the same target protein may also be available for purchase. Users may prefer small-molecule drugs for the convenience of subsequent experimental testing. In the above-mentioned case, users might replace the antibody drug erenumab with the small-molecule drug olcegepant. In summary, the drugs recommended by OTTM are for reference only. Users make the final choice on the drugs to be tested. To ensure clarity of display, OTTM restricts the number of drugs and proteins included in the ECharts sunburst plot and outputs all the recommended drugs and target proteins in another table file.

### Target classification and drug recommendations for interacting proteins

For disease types lacking cellular models, the number of remaining target proteins and recommended drugs might be overwhelming for subsequent experimental validation. Thus, as mentioned above, users can control the number of recommended drugs and target proteins. However, for omics data containing hundreds of candidate proteins or genes, the number of remaining target proteins and recommended drugs may be insufficient for subsequent experimental validation. To provide users with greater flexibility in this situation, OTTM can expand the number of candidate proteins by constructing another protein list consisting of proteins that physically interact with these candidate proteins. OTTM utilizes PPI information from the STRING database to construct this list of interacting proteins [18, 20, 21], which is also regarded as an input list to generate another batch of recommended drugs. OTTM always performs the two-phase target classification and drug recommendation for both the user-provided protein list and this automatically constructed list of interacting proteins. Users decide to adopt or not the recommended drugs from interacting proteins, depending on whether the output number of recommended drugs meets their expectations.

**Figure 4:**
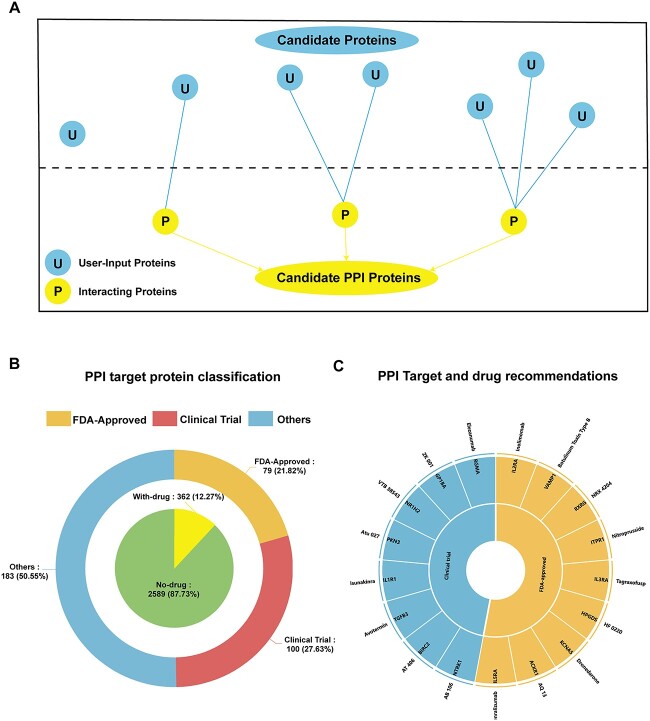
Drugs and target proteins recommended by OTTM for 2951 interacting proteins. (**A**) The principle involved in how OTTM constructs the list of proteins that interact with the user-provided proteins according to PPI information obtained from the STRING database. The cyan circles with uppercase ‘U’ represent candidate proteins provided by users, whereas the yellow circles with uppercase ‘P’ represent interacting proteins. For the 4489 candidate proteins provided, 2951 interacting proteins were identified by OTTM and formed the protein list used for further target classification and drug recommendations. (**B**) Target classification by OTTM of the 2951 interacting proteins based on drug availability assessment. OTTM indicated that 79 interacting proteins have FDA-approved drugs, whereas 100 interacting proteins have drugs undergoing clinical trials. (**C**) From an initial 2951 interacting proteins, 17 drugs and target proteins were recommended by OTTM for further experimental validation, including nine FDA-approved and eight clinical trial drugs. One drug is recommended for each target protein.

The general principle underlying how OTTM constructs this list of interacting proteins is illustrated in Figure 4A. For the initial 4489 candidate proteins, OTTM constructed a list of 2951 interacting proteins based on PPI information from the STRING database. After the two-phase target classification and drug recommendation, OTTM indicated that 2589 (87.73%) of these interacting proteins have no corresponding drug, whereas 362 (12.27%) do. Of these 362 proteins with available drugs, 79 (21.82%) have corresponding FDA-approved drugs, 100 (27.63%) are associated with drugs that are under clinical trial and 183 (50.55%) have been linked to discontinued drugs. Among the 79 interacting proteins with corresponding FDA-approved drugs, nine (11.39%) have not been reported as being relevant to hepatocellular carcinoma, whereas 70 (88.61%) have. Of the 100 proteins associated with clinical trial drugs, eight (8%) have not been reported as being relevant to hepatocellular carcinoma, whereas 92 (92%) have (Supplementary Figure S1). Finally, 17 drugs were recommended by OTTM for the automatically constructed list of 2951 interacting proteins. These drugs, together with the 23 recommended drugs obtained from the 4489 user-provided candidate proteins, are expected to be evaluated in subsequent experimental testing. However, only 15 of these 40 drugs (detailed information is provided in Supplementary Table S7) were available for purchase, and these were subsequently tested on the Hep-G2 hepatocellular carcinoma cell line.

### Evaluation of the anticancer effects of the recommended drugs

**Figure 5:**
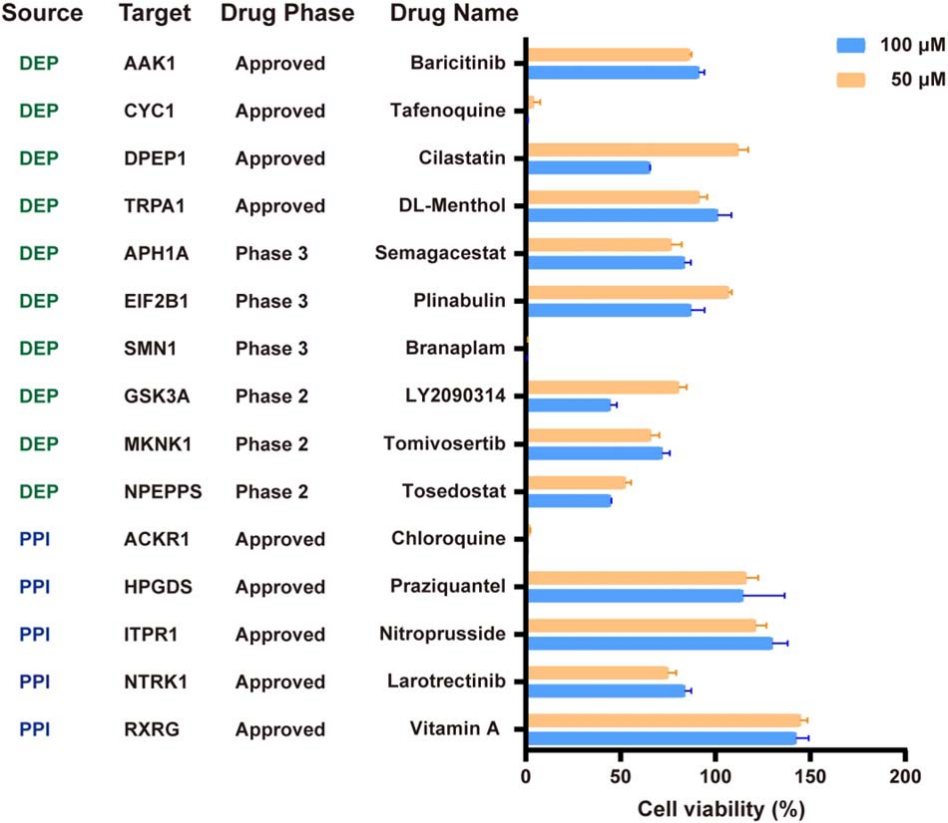
Cell viability assay for the 15 commercially available drugs recommended by OTTM. Combined, OTTM recommended 40 drugs, including 23 for the 4489 candidate proteins and 17 for the 2951 interacting proteins. Among the 40 drugs recommended by OTTM, 15 were commercially available and were tested for their activity against the HepG2 hepatocellular carcinoma cell line. The target proteins of these 15 drugs were classified into two categories by source. DEP represents the DEPs identified from the omics data and PPI represents the interacting proteins according to existing PPI information from the STRING database.

To evaluate the anticancer activity of the 15 drugs, we assessed the viability of Hep-G2 and HuH-7 cells following treatment with 50 and 100 μM concentrations of these drugs (Figure 5 and Supplementary Figure S3). Five drugs showed more than 50% inhibition against Hep-G2 cell viability at the concentration of 100 μM. Subsequently, the IC_50_ values of these five drugs against Hep-G2 cells were measured via dose–response curve fitting, as shown in Supplementary Figure S2. Of these five drugs, tafenoquine succinate (a FDA-approved antimalarial drug targeting CYC1) and branaplam (a Phase 3 clinical trial drug targeting SMN1 for the treatment of spinal muscular atrophy) have not been reported as being associated with hepatocellular carcinoma [22–24]. The IC_50_ values of tafenoquine succinate and branaplam against the growth of Hep-G2 cells were 3.04 and 0.12 μM, respectively. These drugs have either been approved by the FDA or are undergoing clinical drugs for non-cancer indications, which facilitate their experimental validation. Furthermore, their inhibitory effects on Hep-G2 cell proliferation suggest that their corresponding target proteins, CYC1 and SMN1, may be critical for the survival of hepatocellular carcinoma cells [25–27].

## DISCUSSION AND CONCLUSION

Hundreds and even thousands of DEPs or DEGs are typically identified from omics data. For translational medicine research from omics data, candidate proteins with corresponding drugs, especially those approved by the FDA or undergoing clinical trials, are preferred for subsequent drug efficacy assessment and further target validation [28–31]. Most candidate proteins identified from omics data have no corresponding drugs or even compounds. It is inefficient to manually check drug availability for thousands of candidate proteins. Moreover, most candidate proteins with corresponding drugs might already have been reported associated with the disease of interest. Literature mining has proved to be a beneficial approach to drug discovery [12, 13, 32]. However, it is not practical to manually check every literature reference for these candidate proteins. Consequently, it is necessary to develop an efficient classification tool aimed at the two above-mentioned tasks for omics-based translational medicine studies. Such a tool would be expected to identify all the proteins categorized as ‘With Drugs’ as well as ‘Not Reported’ among the thousands of proteins or genes identified from omics data.

**Figure 6:**
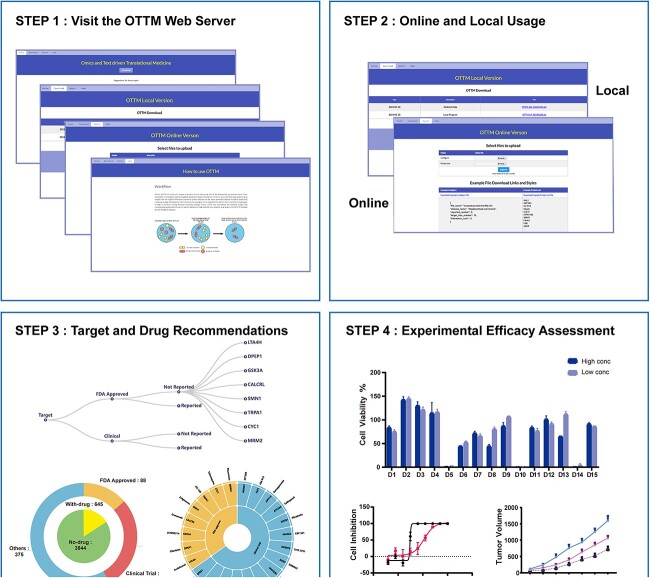
General workflow of OTTM usage. The usage of OTTM includes four steps. Step 1: visit the OTTM web server for online and local usage. Step 2: for local usage, users must download the executable packages and necessary data. For online usage, users must upload a protein list and a configuration file. Step 3: inspect the OTTM-generated output results for recommended drugs. Step 4: purchase the available drugs recommended by OTTM and assess their efficacy in experiments.

In this study, we report the development and exemplary application of a new classification tool, named OTTM, for omics data-driven translational drug discovery. For the thousands of candidate proteins identified from omics data, OTTM performs two-phase classifications, namely, a drug availability assessment with existing drug–target pair information, and literature mining with all available PubMed abstract information. Finally, OTTM retains the candidate proteins categorized as ‘With Drugs’ and ‘Not Reported’ and recommends one drug for each target protein. Dozens of recommended drugs can be assessed for their efficacy against the disease of interest in subsequent experiments (Figure 6). For disease types with cellular models, 10–20 drugs are suggested to be tested, whereas for disease types with only animal models, 5–10 drugs are suggested to be tested. Users can control the number of recommended drugs in the input configuration file of OTTM. As the drugs recommended by OTTM are FDA-approved or under clinical trial, relevant clinical studies can be rapidly launched once the drugs have been confirmed as effective, saving the time and cost of *de novo* drug development [33].

The current version of OTTM has several limitations. First, some disease types might have more than one name. For instance, running OTTM with the keyword ‘hepatocellular carcinoma’ might produce slightly different results compared with running it using the keyword ‘liver cancer’. Users may overcome this limitation by comparing the recommended drugs using different keywords and making comprehensive decisions. Second, the criteria for literature mining are strict, as the keyword designated by users cannot be found in any abstract of the evaluated protein. Some candidate proteins might be categorized as ‘Reported’ and excluded by OTTM owing to a coincidental appearance in the same abstract as the designated keyword. A later version of OTTM may improve this aspect via the introduction of more intelligent methods. Third, some recommended drugs may be antibodies. For target proteins that only have an antibody as the corresponding drug, this recommendation is acceptable. However, for target proteins associated with both antibody and small-molecule drugs, most users may prefer the latter. OTTM cannot distinguish small-molecule drugs from antibody drugs by name. To overcome this limitation, the drug–target pair information used by OTTM can undergo further classification in a later version.

An exemplary application was carried out in this study using our previously obtained proteomics and transcriptomics data from clinical samples of hepatocellular carcinoma. From 4489 candidate proteins identified from these omics data, only 23 target proteins were retained by OTTM for subsequent experimental validation. These 23 target proteins were categorized as both ‘With Drugs’ and ‘Not Reported’, thus meeting the requirement for inclusion in subsequent translational medicine studies. This marked decrease in scale is accomplished via two phases of target classification. Following the drug availability assessment, 3844 (85.63%) candidate proteins were excluded, whereas an additional 622 (13.86%) candidate proteins were excluded after literature mining. Combined, OTTM excluded 4466 candidate proteins, which was more than 99% of the total. Thus, OTTM greatly narrows down the number of drugs and targets for further experimental investigation. The time and effort needed to manually exclude these 99% of proteins is considerable; however, this process is accomplished by OTTM in several minutes on typical personal computers, indicating that OTTM has a low requirement for computer hardware resources. It is worth noting that the candidate proteins without corresponding drugs may also be relevant to the disease of interest. While their roles are to be explored using other complementary tools that focus on proteins without drugs or even compounds.

There are obvious differences between OTTM and existing computational tools or platforms for the studies of translational drug discovery and target discovery [14, 32, 34, 35]. The prominent difference between OTTM and most existing tools is that OTTM does not make a putative judgment on whether a protein or a drug might be correlated with the disease of interest [36]. This correlation is uncovered through subsequent experimental assessment of drug efficacy. In other words, OTTM pays more attention on the target and drug space unexplored in the literature, to guarantee the scientific novelty of subsequent experimental discovery. Once a drug or several drugs recommended by OTTM are found to be effective against the disease of interest, users will assume that their corresponding target proteins are important to the disease and are worth investigating. Therefore, subsequent experimental testing and validation are indispensable to the usage of OTTM.

Compared with experimental drug discovery strategies, OTTM has marked advantages in terms of efficiency. First, in traditional studies of translational drug discovery, only one drug, or one class of drugs, is typically discovered from a batch of omics data. However, a batch of omics data likely contains multiple effective drugs of different types and indications. Thus, OTTM is expected to improve the efficiency of translational drug discovery from omics data. Second, when compared with experimental screening using only FDA-approved drug libraries, which are commercially available from chemical companies, typically more than half of the drugs recommended by OTTM are under clinical trial. This difference provides greater exploration space for translational medicine research and means that using OTTM is more likely to result in the discovery of novel effective drugs and target proteins for diseases of interest.

**Key Points**

- Omics and Text driven Translational Medicine (OTTM) is a new classification tool for identifying all ‘With Drug’ and ‘Not Reported’ targets from up to thousands of proteins or genes identified from omics data.
- OTTM can greatly narrow down the range of candidate proteins and drugs for testing to dozens via drug availability assessment and literature mining.
- OTTM guarantees that the target proteins for the drugs to be tested have not been previously reported as being relevant to the disease of interest.
- Drugs recommended by OTTM are FDA-approved or under clinical trial. Once proven effective in efficacy assessment, clinical trials can speedily be launched.

## Supplementary Material

Supplementary_Tables_of_OTTM_0722_bbad301

Sup_OTTM_0722_bbad301

## Data Availability

Source codes and data of OTTM are available in the GitHub repository (https://github.com/YXB-OTTM/OTTM). Experimental data are incorporated into the article and its online supplementary material.

## Notes

**Xiaobo Yang** is an MS student at ShanghaiTech University. His research interest is tools development for drug and target discovery using bioinformatics. His affiliation is the School of Life Science and Technology, ShanghaiTech University, 393 Huaxiazhong Road, Shanghai 200031, China.

**Bei Zhang** is a PhD student at Shanghai Institute of Materia Medica. Her research interest is drug and target discovery using new strategies and tools. Her affiliation is the Drug Discovery and Design Center, Shanghai Institute of Materia Medica, Chinese Academy of Sciences, 555 Zuchongzhi Road, Shanghai 201203, China; University of Chinese Academy of Sciences, No.19A Yuquan Road, Beijing 100049, China.

**Siqi Wang** is an MS student at Beijing Proteome Research Center. Her research interest is drug and target discovery from collected proteomics data. Her affiliation is the State Key Laboratory of Proteomics, Beijing Proteome Research Center, and National Center for Protein Sciences (Beijing).

**Ye Lu** is an MS student at Nanjing University of Chinese Medicine. Her research interest is drug and target discovery using new strategies and tools. Her affiliation is the School of Chinese Materia Medica, Nanjing University of Chinese Medicine, Nanjing 210023, China; Chemical Biology Research Center, Shanghai Institute of Materia Medica, Chinese Academy of Sciences, Shanghai 201203, China.

**Kaixian Chen** is an academician medicinal scientist of the Chinese Academy of Sciences. His research interest is methods and tools development for drug discovery and drug design. His affiliation is the Drug Discovery and Design Center, Shanghai Institute of Materia Medica, Chinese Academy of Sciences, 555 Zuchongzhi Road, Shanghai 201203, China; University of Chinese Academy of Sciences, No. 19A Yuquan Road, Beijing 100049, China; School of Life Science and Technology, ShanghaiTech University, 393 Huaxiazhong Road, Shanghai 200031, China; School of Chinese Materia Medica, Nanjing University of Chinese Medicine, Nanjing 210023, China.

**Cheng Luo** is a professor at Shanghai Institute of Materia Medica. His research interest is chemical biology research using interdisciplinary technologies. His affiliation is the Chemical Biology Research Center, State Key Laboratory of Drug Research, Shanghai Institute of Materia Medica, Chinese Academy of Sciences, 555 Zuchongzhi Road, Shanghai 201203, China; University of Chinese Academy of Sciences, No. 19A Yuquan Road, Beijing 100049, China; School of Chinese Materia Medica, Nanjing University of Chinese Medicine, Nanjing 210023, China.

**Aihua Sun** is aassociate professor at Beijing Proteome Research Center. Her research interest is translational medicine research using proteomics data. Her affiliation is the State Key Laboratory of Proteomics, Beijing Proteome Research Center, National Center for Protein Sciences (Beijing), Beijing Institute of Lifeomics; Research Unit of Proteomics-driven Cancer Precision Medicine, Chinese Academy of Medical Sciences.

**Hao Zhang** is an associate professor at Shanghai Institute of Materia Medica. His research interest is tool development and application research using bioinformatics and drug design technologies. His affiliation is the Chemical Biology Research Center, State Key Laboratory of Drug Research, Shanghai Institute of Materia Medica, Chinese Academy of Sciences, 555 Zuchongzhi Road, Shanghai 201203, China.
