# Supplementary material for: OTTM: an automated classification tool for translational drug discovery from omics data
Source: Brief Bioinform. 2023 Aug 18;24(5):bbad301. doi: 10.1093/bib/bbad301 (PMC10516341; doi:10.1093/bib/bbad301)
Supplement: Sup_OTTM_0722_bbad301 [file sup_ottm_0722_bbad301.docx]

**Supplementary Information for**

**OTTM: An automated classification tool for translational drug discovery from omics data**

Supplementary Figure S1

Supplementary Figure S2

Supplementary Figure S3


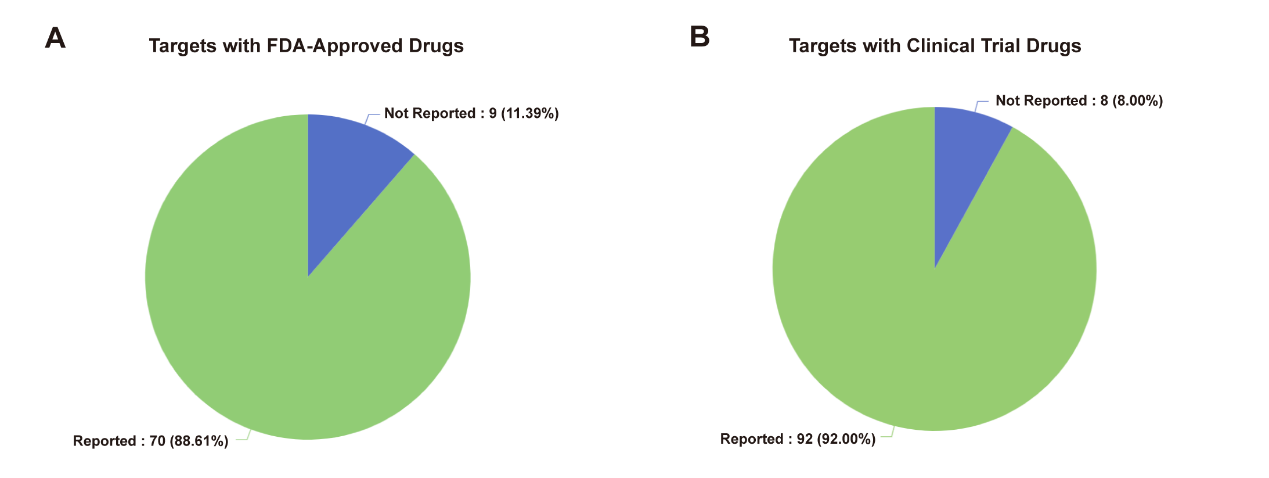


**Supplementary Figure S1. Target classification via literature mining for PPI proteins with available drugs.**

**(A)** Among the 79 PPI proteins with FDA-approved drugs, OTTM suggests that 9 PPI proteins have not been reported relevant to hepatocellular carcinoma, while the other 70 target proteins have at least one PubMed abstract containing the designated keyword “hepatocellular carcinoma”.

**(B)** Among the 100 PPI proteins with clinical trials drugs, OTTM suggests that 8 PPI proteins have not been reported relevant to hepatocellular carcinoma, while the other 92 target proteins have at least one PubMed abstract containing the designated keyword “hepatocellular carcinoma”.


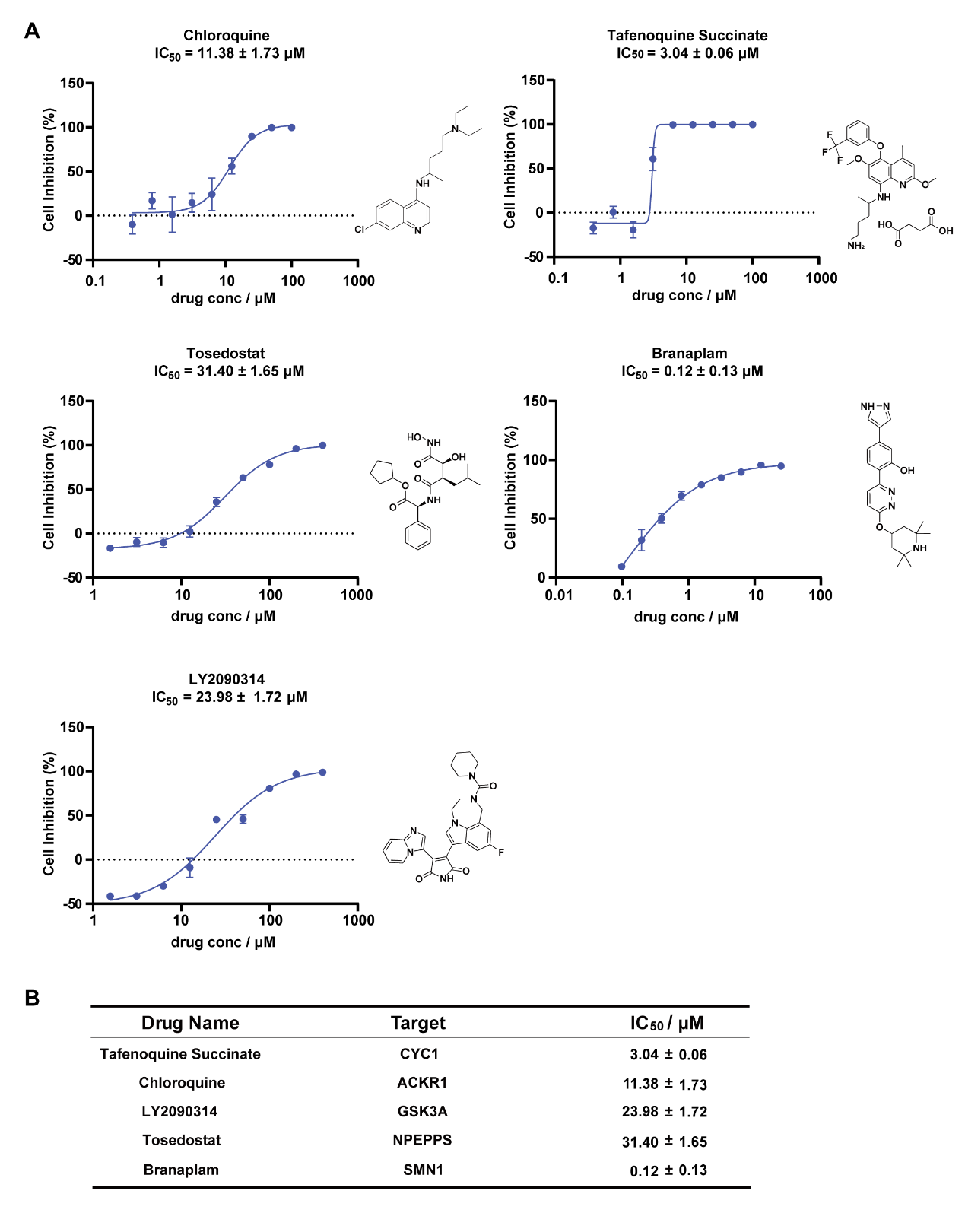


**Supplementary Figure S2. IC_50_ curves and values for 5 drugs with obvious inhibitions against hepatocellular carcinoma Hep-G2 cells.**

**(A)** IC_50_ curves and fitted values for 5 drugs with obvious inhibitions against Hep-G2 cells.

**(B)** IC_50_ values and target information summary for 5 drugs tested.

**
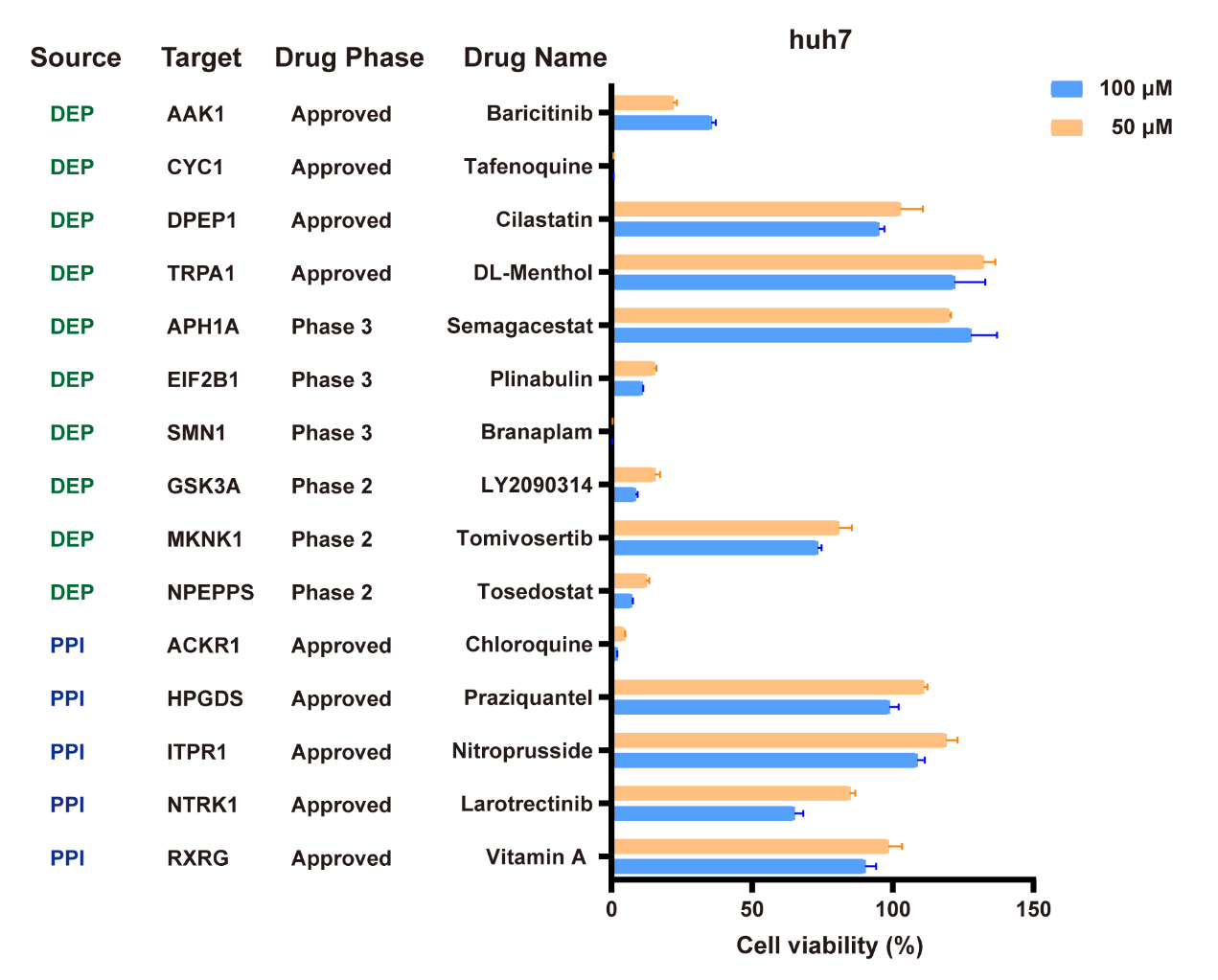
**

**Supplementary Figure S3. Cell viability assay for the 15 commercially available drugs recommended by OTTM on the HuH-7 cell line.**

The 15 commercially available drugs were tested for their activity against the Huh-7 hepatocellular carcinoma cell line. DEP represents the differentially expressed proteins identified from the omics data and PPI represents the interacting proteins according to existing protein-protein interaction information from the STRING database.
